# Letter to the editor: A critical look at the surgical management of appendiceal goblet-cell adenocarcinoma

**DOI:** 10.1016/j.ijscr.2025.111912

**Published:** 2025-09-04

**Authors:** Parth Aphale, Shashank Dokania, Himanshu Shekhar

**Affiliations:** Dr. D.Y. Patil Vidyapeeth (Deemed to be University), Pimpri, Pune, Maharashtra, India

Dear Editor,

We read with great interest the case report by Xie and Li on a chance diagnosis of appendiceal goblet-cell adenocarcinoma (GCA) treated by ileocecectomy plus partial right-hemicolectomy [1]. We appreciate the authors' effort in presenting a rare case that highlights the challenges in managing this unique malignancy. We concur that the diagnosis of GCA is often incidental and presents a significant clinical dilemma, particularly regarding the extent of surgical resection [1]. However, we wish to offer a constructive critique and raise several points for discussion.

The case report's central argument rests on the appropriateness of ileocecectomy plus partial right-hemicolectomy as a definitive treatment based on preoperative imaging studies showing no metastasis. While this approach was successful in this specific case, it may not be universally applicable and could represent a more conservative management strategy that diverges from the current standard of care for many institutions. The consensus guidelines and recent literature continue to advocate for a right hemicolectomy as the standard treatment for GCA, even in cases without apparent metastasis, due to the tumor's aggressive biological behavior and potential for microscopic spread that is undetectable by conventional imaging. Although the authors note that ileocecectomy plus partial right-hemicolectomy was chosen based on specific preoperative evaluation, this decision-making process warrants further clarification. What specific metrics or findings from the enhanced CT and colonoscopy were so convincing as to deviate from the established norm? The article mentions that a negative CT and colonoscopy were a basis for this choice, but what about other important factors? The presence of perineural invasion in the final pathology report, which the authors themselves noted, is a known poor prognostic factor that increases the risk of recurrence and systemic spread, and often dictates a more aggressive surgical approach and consideration for adjuvant therapy. The final pathological staging of pT4aN0M0 also indicates that the tumor invaded the visceral peritoneum, a feature that many guidelines would consider a strong indication for a more extensive resection [[2], [3], [4]].

The authors also touch upon the difficulty of avoiding tumor cell spillage during the initial appendectomy, which is a critical concern that they rightly point out lacks sufficient data. This is a pivotal point that deserves more emphasis and discussion. While a plastic retrieval bag was used, the potential for microscopic spillage remains a major risk factor for peritoneal metastasis. This brings to light the larger question of whether appendectomy alone is ever a safe initial procedure for suspected appendiceal tumors, and if not, what preoperative markers or imaging features should prompt a more cautious approach [5].

In conclusion, while we commend the authors for their contribution, we feel that the article could be improved by a more detailed justification of the surgical decision-making process. Future studies should focus on establishing clearer criteria for a less extensive resection than a full right hemicolectomy and explore the long-term oncological outcomes of such an approach in patients with risk factors like perineural invasion.

## Author contribution

**SD:** Writing – original draft, Writing – review & editing. **HS:** Supervision, Writing – review & editing. **PA:** Conceptualization, Writing – review & editing.

## Consent

Not applicable.

## Ethical approval

This manuscript is a letter to the editor, hence ethical approval is not applicable.

## Guarantor

Dr. Parth Aphale.

## Research registration number

None.

## Funding

This manuscript did not receive any funding from any source.

## Conflict of interest statement

The authors declare that they have no known competing financial interests or personal relationships that could have appeared to influence the work reported in this paper.

## Data Availability

No data was used for the research described in the article.
